# Identification of New Drug Target in *Staphylococcus lugdunensis* by Subtractive Genomics Analysis and Their Inhibitors through Molecular Docking and Molecular Dynamic Simulation Studies

**DOI:** 10.3390/bioengineering9090451

**Published:** 2022-09-07

**Authors:** Yahya Alhamhoom, Umme Hani, Fatima Ezzahra Bennani, Noor Rahman, Md Abdur Rashid, Muhammad Naseer Abbas, Luca Rastrelli

**Affiliations:** 1 Department of Pharmaceutics, College of Pharmacy, King Khalid University, Abha 62529, Saudi Arabia; 2 Laboratory of Pharmacology and Toxicology, Bio Pharmaceutical and Toxicological Analysis Research Team, Faculty of Medicine and pharmacy, Mohammed V University in Rabat, BP6203 Rabat, Morocco; 3 Laboratory of Medicinal Chemistry, Faculty of Medicine and pharmacy, Mohammed V University in Rabat, BP6203 Rabat, Morocco; 4 Department of Biochemistry, Abdul Wali Khan University Mardan, Mardan 23200, Pakistan; 5 Department of Pharmacy, Kohat University of Science and Technology (KUST), Kohat 26000, Pakistan; 6 Dipartimento di Farmacia, University of Salerno, Via Giovanni Paolo II, 84084 Fisciano, Italy

**Keywords:** drug targets, subtractive genomics, homology modeling, molecular docking, molecular dynamics simulation

## Abstract

*Staphylococcus lugdunensis* is a coagulase-negative, Gram-positive, and human pathogenic bacteria. *S. lugdunensis* is the causative agent of diseases, such as native and prosthetic valve endocarditis, meningitis, septic arthritis, skin abscesses, brain abscess, breast abscesses, spondylodiscitis, post-surgical wound infections, bacteremia, and peritonitis. *S. lugdunensis* displays resistance to beta-lactam antibiotics due to the production of beta-lactamases. This study aimed to identify potential novel essential, human non-homologous, and non-gut flora drug targets in the *S. lugdunensis* strain N920143, and to evaluate the potential inhibitors of drug targets. The method was concerned with a homology search between the host and the pathogen proteome. Various tools, including the DEG (database of essential genes) for the essentiality of proteins, the KEGG for pathways analysis, CELLO V.2.5 for cellular localization prediction, and the drug bank database for predicting the druggability potential of proteins, were used. Furthermore, a similarity search with gut flora proteins was performed. A DNA-binding response-regulator protein was identified as a novel drug target against the N920143 strain of *S. lugdunensis*. The three-dimensional structure of the drug target was modelled and validated with the help of online tools. Furthermore, ten thousand drug-like compounds were retrieved from the ZINC15 database. The molecular docking approach for the DNA-binding response-regulator protein identified ZINC000020192004 and ZINC000020530348 as the most favorable compounds to interact with the active site residues of the drug target. These two compounds were subjected to an MD simulation study. Our analysis revealed that the identified compounds revealed more stable behavior when bound to the drug target DNA-binding response-regulator protein than the apostate.

## 1. Introduction

*Staphylococcus lugdunensis* is a coagulase-negative and Gram-positive staphylococci first identified in 1988 [1]. *S. lugdunensis* is usually present on human and other mammals’ skin [2]. Meningitis, septic arthritis, skin abscesses, brain abscess, breast abscesses, peritonitis, spondylodiscitis, and wound infections are different diseases associated with *Staphylococcus lugdunensis* [3]. *S. lugdunensis* is the causative agent of bacteremia in some patients [4]. Most virulent factors of *Staphylococcus lugdunensis* are identical to *staphylococcus aureus* [5]. Both bacteria share approximately 70% of the genome [6]. For *S. lugdunensis* identification, the ornithine decarboxylase test is mainly used [7]. The clinical properties, infection, and biochemical features of *S. lugdunensis* are identical to *S. aureus* [8]. Genome sequencing flaunts the genes encoded for virulency, such as hemolysin, adhesions, and toxins [9]. Biofilm production is an essential factor contributing to the pathogenicity of *Staphylococcus*. Due to the presence of the *ica* gene, *S. lugdunensis* has the ability of biofilm formation [10]. The pathogen becomes methicillin-resistant when SCC mec or staphylococcal cassette chromosome, a movable genetic element, is transferred to different staphylococcal species [11]. *S. lugdunensis* displays predominant resistance to beta-lactam antibiotics because of the production of beta-lactamase enzymes. A total of 73.3% of *S. lugdunensis* isolates were resistant to ampicillin, and 46.6% to cloxacillin and oxacillin. Resistance to beta-non-lactam antibiotics was lower than beta-lactam antibiotics at 53% for azithromycin and 46.6% for amikacin [12]. Yen and his coworkers stated that *S. lugdunensis* showed 87% resistance to penicillin and 20% to oxacillin [13].

Subtractive genome analysis plays a vital role in drug target identification. Drug targets must be necessary for pathogen survival. In three types of cases, subtractive genomics can be widely used: firstly, for pathogens with no virulent factor identified; secondly, for pathogens resistant to drugs; and thirdly, for pathogens for which no powerful drugs are available. There is a need to develop new drugs and cure diseases as the available drugs for treating diseases caused by different pathogens exhibit side effects, and drug-resistant strains increase daily. A unique therapeutic target in the pathogen is predicted using subtractive genome analysis because the target should only be present in the pathogen, preventing drug interactions within human proteins. Experimental approaches are costly, time-consuming, and provide infrequent results. The subtractive genomics approach is highly effective, quick, and inexpensive, so computational approaches are usually preferred over experimental approaches [14,15]. Drug and vaccine targets can be predicted [16]. Many researchers have utilized the subtractive genomics approach for drug target prediction in *Bacillus anthracis* [17], *Burkholderia pseudomalleii* [18], and *Staphylococcus aureus* [19]. This study aims to identify new, non-homologous, essential, and non-gut flora therapeutic targets in *S. lugdunensis* strain N920143 using subtractive genomics. Additionally, it aims to uncover novel drug target inhibitors and construct a homology model of computationally predicted drug targets.

## 2. Materials and Methods

### 2.1. Pathogen Complete Protein Sequences Retrieval

The complete protein sequences of *Staphylococcus lugdunensis* strain N920143 were downloaded from the NCBI (The National Center for Biotechnology Information; Available online: https://www.ncbi.nlm.nih.gov/ (accessed on 22 May 2022) database [20]. Proteins in the FASTA (Fast Alignment Sequence Test for Application; Available online: https://www.ebi.ac.uk/Tools/sss/fasta/ (accessed on 22 May 2022) were selected for further analysis.

### 2.2. Duplicate Proteins Identification

Proteins possessing ≥ 60% similarity were counted as duplicates. Proteins with amino acid sequences < 100 and duplicates were excluded because they were potentially not essential for the survival of pathogens [21]. The CD-hit program; Available online: http://weizhong-lab.ucsd.edu/cdhit_suite/ (accessed on 8 August 2022) was used to identify duplicate proteins [22].

### 2.3. Non-Paralogs Proteins Identification

The non-paralog set of proteins was submitted to the NCBI BLASTp (Basic Local Alignment Search Tool; Available online: https://blast.ncbi.nlm.nih.gov/Blast.cgi?PAGE=Proteins; (accessed on 8 August 2022), in contrast to homo-sapiens proteins, which used a 10^−4^ E value (expected value) [23]. Only non-homologous proteins were used for further analysis, while human homologous proteins were removed.

### 2.4. Essential Proteins Identification

Key cellular functions of microbes are maintained by vital genes and are therefore considered vital for pathogen survival. Non-homologous protein sequences were submitted to the BLASTp against DEG (differentially expressed genes; Available online: https://tubic.org/deg/public/index.php; (accessed on 8 August 2022) using the anticipated E value of 10^−5^ to predict the pathogen genes involved [24]. The DEG database was used to identify essential genes of the *S. lugdunensis*.

### 2.5. Analysis of Standard and Unique Pathways

Using the KAAS (KEGG Automatic Annotation Server) at the KEGG (Kyoto Encyclopedia of Genes and Genomes) [25], metabolic pathways of *S. lugdunensis* strain N920143 were analyzed. Metabolic pathways of pathogens and humans were identified and compared manually. Pathways that appeared only in the pathogen genome were considered unique to *S. lugdunensis*. In contrast, pathways that appeared in both pathogens and humans were considered common pathways, according to the KEGG database [26].

### 2.6. Proteins Localization Prediction

Identifying the subcellular location of proteins is vital for effective drug target identification. Extracellular cell wall, cytoplasmic, and membrane proteins were differentiated by CELLO V.2.5 (Available online: http://cello.life.nctu.edu.tw/; (accessed on 8 August 2022). Extracellular or membrane proteins could be possible vaccine targets, while cytoplasmic proteins are considered potential drug targets.

### 2.7. Virulent Proteins Identification

PAIDB v2.0 (Available online: http://www.paidb.re.kr; (accessed on 8 August 2022) was used for the identification of virulent proteins. The PAIDB gives essential information about predicted pathogenicity in the prokaryotic genome [27].

### 2.8. Drug Ability Potential of Short-Listed Proteins

The drug bank database (Available online: https://go.drugbank.com/; (accessed on 8 August 2022) was accessed to evaluate the druggability potential of all the selected protein sequences of *S. lugdunensis* strain N920143. FDA-approved drug targets for drug IDs are present in drug bank databases [28].

### 2.9. Gut Metagenome Screening

Gut microbiota is constituted of beneficial bacteria that inhabit the human digestive tract. In the human intestine, almost a hundred trillion microbes exist [29]. Between gut flora and humans, a mutualistic and symbiotic relationship exists [30]. Functions such as inhibiting pathogen growth, enhancing the immune system, and producing energy by fermenting undigested carbohydrates are performed by beneficial microbes in the host [31]. The human microbiome project database (Available online: https://hmpdacc.org/; (accessed on 8 August 2022) was used, and proteins resembling gut flora were excluded [32].

### 2.10. 3D Structure Prediction

As the crystal structure of the drug target was not available in the PDB database (Available online: http://www.rcsb.org/pdb/; (accessed on 8 August 2022), homology modeling was performed. The Phyre2 server can develop a highly confident 3D structure: the Phyre2 online server was used in this study to build 3D structures of the drug target. Sequences of the target protein were taken in FASTA format from the NCBI, and submitted as input files to the Phyre2 server. The 3D structure was developed automatically and finally downloaded in PDB format.

### 2.11. Model Validation

The 3D structures developed via the Phyre2 server were validated by Procheck (Available online: https://www.ebi.ac.uk; (accessed on 8 August 2022) and ProSA web (Available online: https://prosa.services.came.sbg.ac.at; (accessed on 8 August 2022) servers. The Procheck server was used for Ramachandran plot analysis [33]. ProSA web is an online server that performs statistical analysis of protein structures [34].

### 2.12. Molecular Docking

In MOE (Molecular Operating Environment, 2016; Available online: https://www.chemcomp.com/Products.htm; (accessed on 8 August 2022) software, the study of molecular docking was performed to find the hits that interact strongly with the predicted drug target. A default value of 0.05 was used for energy minimization, and the model was protonated. The active site of the predicted drug target protein was identified using the site finder option in MOE. Different parameters like Refinement, Rigid Receptor, Rescoring, London dG, Placement, and Triangle Matcher functions in the MOE dock options, were used [24]. A total of 10,000 drug-like compounds retrieved from the ZINC15 database (Available online: https://zinc15.docking.org/; (accessed on 8 August 2022) were docked with the active site of the drug target.

### 2.13. MD Simulation Study

The effect of the top two hits on the structure and stability of the identified drug target proteins was investigated using molecular dynamics simulation. The Amber 20 software (Available online: https://ambermd.org/; (accessed on 8 August 2022) was used to investigate the dynamics of the drug target in the presence and absence of ligands. Cleaning each structure was the first step for MD simulation. After that, a cubic simulation cell with a periodic boundary condition was built. The (AMBER14) force field was used for the protein. Then, a cubic box of TIP3 water with a box dimension of 12 A^o^ of protein was used. The counter ions (Na^+^ and Cl) were added to neutralize the systems [35,36]. Energy minimization was performed on the systems in 5000 stages, using 2500 steepest-descent steps and 2500 conjugate gradient steps. The systems were gently heated from 0 to 325 Kelvin. The Langevin dynamics approach (1 atm pressure and 310 K temperature) was used as a Langevin thermostat [37]. The MD simulation was run for 100 nanoseconds at a constant temperature of 325 K. Origin software was used for data visualization and analysis.

### 2.14. Post MD Analysis

The conformational changes during simulations were analyzed in this study. The CPPTRAJ module of AMBER 20 was used to investigate the root-mean-square deviation (RMSD), root-mean-square fluctuation (RMSF), and radius of gyration (RoG).

## 3. Results and Discussion

**Figure 1:**
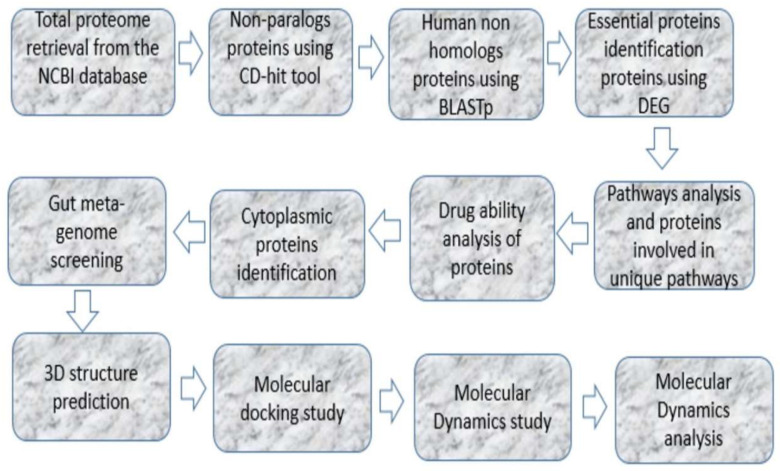
The proposed procedure and methodology followed in the present study.

A new drug target in the *S. lugdunensis* genome was identified by performing subtractive genomics. Figure 1 describes the systematic workflow, and Table 1 describes the relative number of proteins obtained from each step. By subtractive genome analysis, a DNA-binding response-regulator protein was found as a novel drug target in *S. lugdunensis* bacteria. Due to the lack of its 3D structure in the protein databank database (PDB), homology modeling was performed to predict new inhibitors against computationally predicted drug-target-protein molecular docking.

### 3.1. Retrieval of Pathogen Proteome and Removal of Duplicates

Among all the available strains of *S. lugdunensis,* N920143 was selected as this strain was human pathogenic. From the NCBI database, a total of 2240 proteins of *S. lugdunensis* strain N90143 were downloaded. All the protein sequences obtained from the NCBI database were submitted to the CD-hit tool, with an identity value of 60%, to remove duplicate proteins and sequences with fewer than 100 amino acids [21,22]. The total number of proteins of N920143 were minimized to 2102 after running the CD-hit tool.

### 3.2. Pathogen Essential and Non-Homologous Genes Identification

Non-duplicate proteins were submitted to the NCBI BLASTp (Available online: https://blast.ncbi.nlm.nih.gov/Blast.cgi?PAGE=Proteins; (accessed on 8 August 2022) against human proteome using a 10^−4^ E value [38]. A total of 980 proteins were found as non-homologous in the N920143 strain. The basic standard for potent drug targets is that drug target proteins are crucial for pathogen survival, but must be absent in the human host so that human and pathogen proteins do not cross-bind and to avoid the side effects of drugs [15]. Complete protein sequences of the N920143 strain were submitted to the BLASTp instead of a DEG (database of essential genes) to determine the pathogen’s essential genes, using expected values 10^−5^. In the N920143 strain, 670 essential proteins were found in the database of essential genes.

### 3.3. Pathways Analysis

The KEGG database was used to analyze the pathogen and host metabolic pathways through the KAAS server [39]. Common pathways were not considered, and only the pathogen’s unique pathways were preferred. In the N920143 strain of *S. lugdunensis*, 21 unique pathways were found. Unique pathways, along with pathway IDs, are presented in Table 2. In these unique pathways, five proteins were involved (Table 3). A single protein could be involved in more than one pathway. Proteins that are not homologous to humans and take part in multiple pathways can be a more potent drug target [26].

### 3.4. Prediction of Protein Subcellular Localization

According to CELLO V.2.5, 70% of the proteins were present in the cytoplasm and 30% were present in the membrane.

### 3.5. Druggability of Selected Proteins

For the druggable potentiality identification, all the essential and non-homologous proteins were blasted with a drug bank database, containing FDA-approved drug targets. Five proteins revealed similarities with FDA-approved drugs in the drug bank database. Table 4 delineates proteins possessing druggable potency, along with drug bank IDs.

### 3.6. Screening of Short-Listed Proteins with Gut Flora

Along with pathogenic bacteria, the beneficial bacteria in human gut flora can also be targeted by most antibiotics [40], so we tended to exclude those proteins of *S. lugdunensis* that displayed homology with gut flora proteins to avoid the side effects of drugs. Regarding this concept, the human microbiome project database was used to evaluate those proteins that showed similarities with regular gut flora proteins. Of the human non-homologous, virulent, and essential proteins that were compared with gut flora proteins, 11 out of 12 displayed similarities with gut flora. Only one DNA-binding response-regulator protein did not display any similarity with gut flora; therefore, this was selected as a novel drug target in *S. lugdunensis*.

### 3.7. Homology Modeling and Model Validation

**Figure 2:**
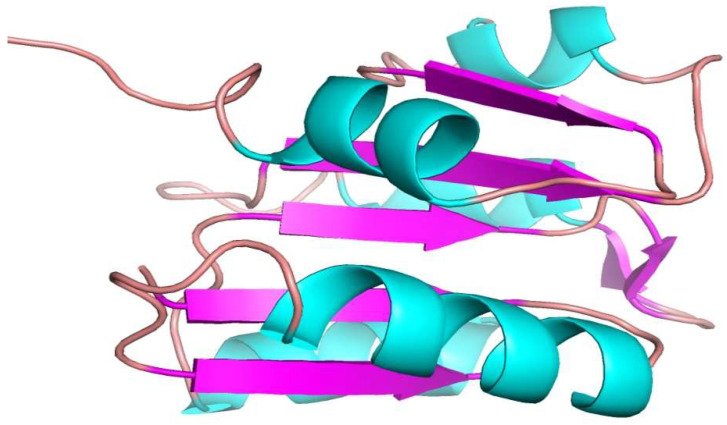
3D structure of the drug target DNA-binding response regulator modeled by Phyre2 server.

**Figure 3:**
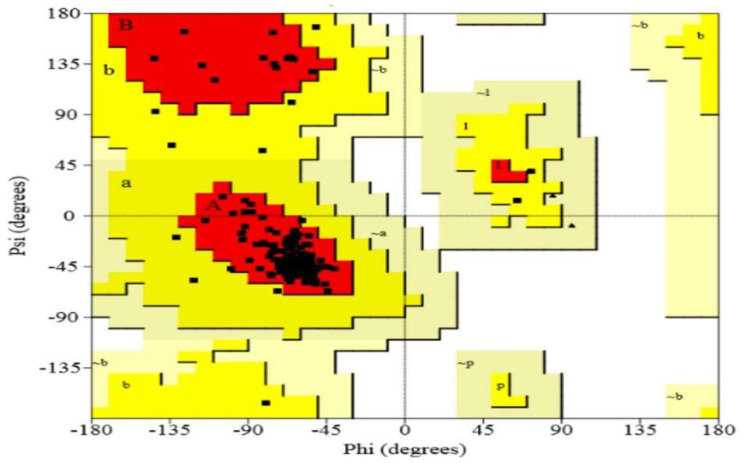
Ramachandran plot for structural validation of drug target protein (DNA-binding response regulator). The most favored zone contains 93.6% of the residues, the additional allowed region contains 6.4%, and the disallowed region contains 0% of the residues. (a = α-helix (right/left handed); B = anti-parallel β-sheet; b = parallel β-sheet; p = proline. The coloring/shading on the plot represents the allowed phi-psi backbone conformational regions, where the darkest areas (in red) correspond to the most favorable combinations of phi-psi values).

**Figure 4:**
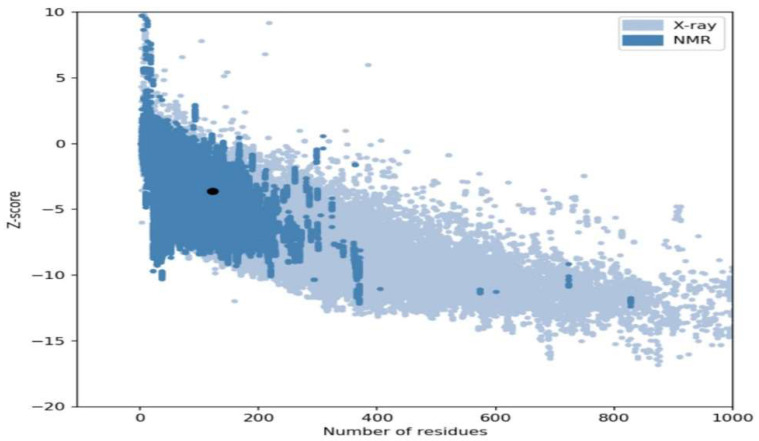
Z-score of the drug target protein (DNA-binding response regulator) is −3.63.

The crystal structure of the target protein was not found in the PDB database; homology modeling was performed via the Phyre2 server. Figure 2 shows the 3D structures of the drug target DNA-binding response regulator. The 3D structure was further validated by the Ramachandran plot and the ProSA web server. The quality of the model was predicated on the Z-score plot of the ProSA web server. The Z-score of the excellent quality model lies within the range of native protein structures, while the erroneous structure has a Z-score outside this range [33]. The Z-score of the target DNA-binding response-regulator protein is −3.63, as in Figure 3. Ramachandran plot analysis was performed by the Procheck server [17]. According to the Ramachandran plot, 94.7% of residues are in the most favored region, 5.3% in the additional allowed region, 0% in the generously allowed, and 0% in the disallowed region as seen in Figure 4. The Ramachandran plot quantified that the quality of the model is good if >90 % of residues are in the most favored regions.

### 3.8. Molecular Docking Study

**Figure 5:**
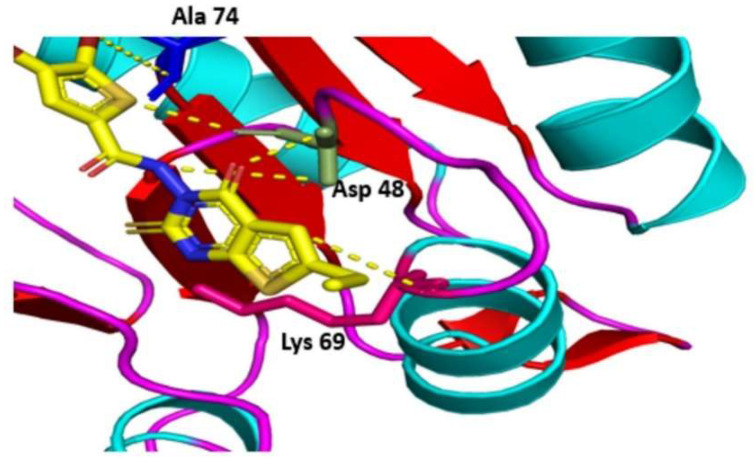
Hydrogen bond interaction of ZINC000020192004 with the drug target.

Ten thousand compounds of zinc database were docked against the receptor active site of the DNA-binding response regulator. Among the top 100 compounds, two hits yielded a favorable interaction with the DNA-binding response-regulator protein (Figure 5). Compounds ZINC000020192004 and ZINC000020530348, with docking scores of −16.231 and −14.187, were found as novel and potent inhibitors against the predicted drug target protein. Docking scores of these potent inhibitors are shown in Table 5.

### 3.9. MD Simulation

**Figure 6:**
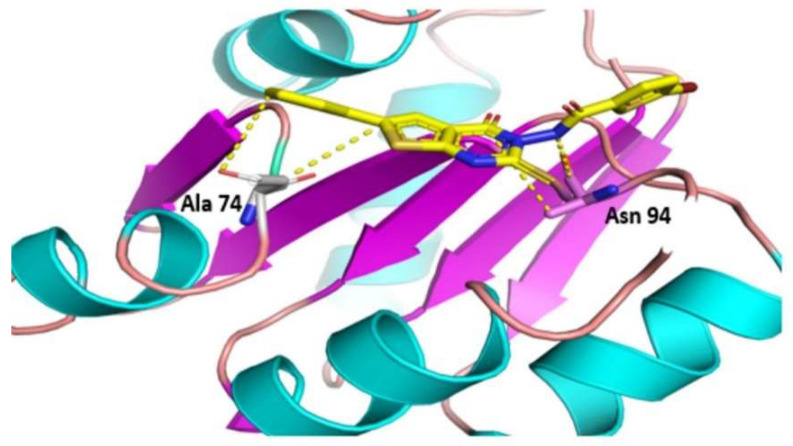
Hydrogen bond interaction of ZINC000020530348 with the drug target.

**Figure 7:**
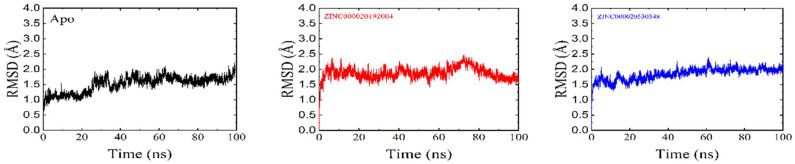
RMSD of apostate (black color) and the ZINC000020192004 and ZINC000020530348.

The RMSD parameter determined the complex stability. The complex is more stable when the average RMSD value is low [41,42] (Figure 6). The RMSD plots for ZINC000020192004 and ZINC000020530348 in complex with drug target and unbound (apo-enzyme) are shown in Figure 7. The two complexes converged at around 5 ns and remained stable during the simulation, with overall average values of 1.6 and 1.5. The apo-enzyme average RMSD is 1.6A.

**Figure 8:**
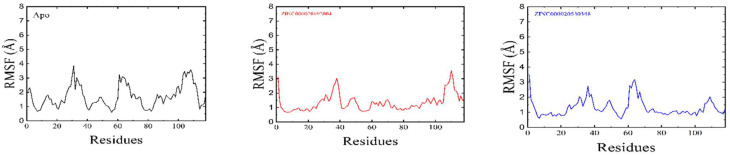
RMSF graphs of the apostate, ZINC000020192004, and ZINC000020530348. The number of residues is shown on the x-axis.

The average RoG values of 16.2, 16.1, and 16.01 were observed for ZINC000020192004 and ZINC000020530348, and the apo-enzyme. The RMSF measures how the residues fluctuate when bound to a drug [43]. An increase in the RMSF value indicates an increase in the flexibility of the alpha-carbon atoms. Compared to the two predicted hits, the more flexible regions were found in the apo-enzyme. High residual fluctuations were recorded at residues 20–40 and 60–80, as shown in Figure 8.

**Figure 9:**
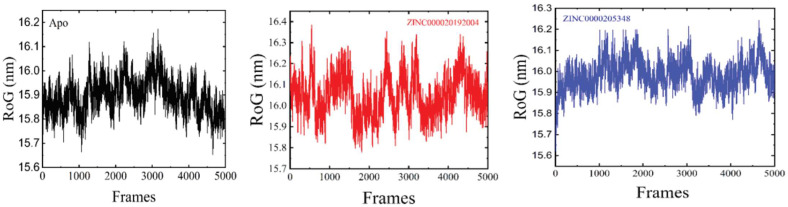
RoG of the apostate, ZINC000020192004 and ZINC000020530348. The number of frames is shown on the x-axis.

The RoG parameter was used to evaluate the structural compactness of proteins when bound to molecules [44]. A lower RoG value indicates more stability, while a higher RoG value indicates an unstable system [45]. The RoG plot results correlate with its RMSD plot, indicating that molecule binding did not affect the structural stability of the DNA-binding response-regulator protein [46]. In the case of the ligand-bound complexes, a lower RoG value was revealed; meanwhile, for the apoenzyme, a slightly higher RoG value was indicated, as is displayed in Figure 9.

## 4. Conclusions

In the present study, we utilized the art of computational biology to identify novel therapeutic targets in *S. lugdunensis* proteome. Through subtractive proteomic analysis, novel drug targets involved in unique metabolic pathways were identified by using comparative sequence analysis and different biological updated databases. The prioritized therapeutic targets, including 4-hydroxtetrahydrodipicolinate reductase, signal peptidase, DNA-binding response regulator, and genome polyprotein, are critical to the pathogen’s survival. The drug target predicted in the present study was promising to be essential to the pathogen survival and did not share homology with the human gut microbiota. The three-dimensional structure of the DNA-binding response regulator-protein was subjected to molecular docking studies, and it evaluated ZINC000020192004 and ZINC000020530348 as lead compounds. These compounds showed striking interactions with the DNA-binding response regulator. Furthermore, the results of the MD simulation analysis demonstrated that the two hits, ZINC000020192004 and ZINC000020530348, remained stable with the active site residues of the DNA-binding response-regulator protein. Further in vitro study is needed to validate these drug targets and the lead compounds.

## Figures and Tables

**Table 1:** The relative number of proteins obtained from each step.

S. No.	Steps Followed	No. of Proteins
1	The total proteome of the N920143 strain downloaded from NCBI	2351
2	Non-paralogous proteins obtained from the CD-HIT tool	2102
3	Human non-homologous proteins obtained from BLASTp against humans	980
4	Proteins essential to pathogen survival obtained from DEG	670
5	Pathways unique to the pathogen	21
6	Proteins involved in pathogen-unique pathways	5
8	Analysis of druggability potential of proteins	5
9	Number of cytoplasmic proteins obtained from CELLO	3
10	Gut metagenome screening	1

**Table 2:** Unique metabolic pathways of *S. lugdunensis*, along with pathway IDs.

S. No.	Pathway	Metabolic Pathway
1	sln00280	Lysine biosynthesis
2	sln00550	Peptidoglycan biosynthesis
3	sln00121	Secondary bile acid biosynthesis
4	sln00053	Ascorbate and aldarate metabolism
5	sln02020	Two-component system
6	sln00261	Monobactam biosynthesis
7	sln01110	Biosynthesis of secondary metabolites
8	sln02024	Quorum sensing
9	sln01210	2-Oxocarboxylic acid metabolism
10	sln00460	Cyanoamino acid metabolism
11	sln00622	Xylene degradation
12	sln01220	Degradation of aromatic compounds
13	sln00450	Selenocompound metabolism
14	sln01501	beta-Lactam resistance
15	sln00521	Streptomycin biosynthesis
16	sln03070	Bacterial secretion system
17	sln00860	Porphyrin and chlorophyll metabolism
18	sln01502	Vancomycin resistance
19	sln01503	CAMP resistance
20	sln00660	Biosynthesis of siderophore group non-ribosomal Peptides
21	sln01120	Microbial metabolism in diverse environments

**Table 3:** Proteins involved in unique pathways.

S. No.	Accession No.	Drug Bank Target	Drug Bank ID
1	WP_002460335.1	P0A6K3 Peptide deformylase	DB01942
2	WP_014533179.1	P04217 Alpha-1B-glycoprotein	DB01593
3	WP_002459785.1	P17405 Sphingomyelin phosphodiesterases	DB01151
4	WP_002491992.1	Q13231 Chitotriosidase-1	DB02325
5	WP_002459785.1	P17405 Sphingomyelin phosphodiesterase	DB01151

**Table 4:** Non-homologous, essential, and virulent druggable targets of *S. lugdunensis*.

S. No.	Accession No.	KEGG ID	Target Name	Pathway Name
1	WP_002478208.1	K00215	4-hydroxtetrahydrodipicolinate reductase	Monobactam biosynthesis
2	WP_002461066.1	K03100	Signal peptidase	Quorum sensing
3	WP_002460335.1	K07705	DNA-binding response regulator	Two-component system
4	WP_026050227.1	K06153	Genome polyprotein	Bacterial secretion system
5	WP_011079778.1	K02034	Chloride channel protein 2	beta-lactam resistance

**Table 5:** Molecular docking of the most favorable interacting compounds.

S. No.	ZINC ID	S Score	Interacting Residues	Energy
1	ZINC000020192004	−16.231	ALA 74 ASP 48 ALA 74 LYS 69 ALA 74	−5.0 −1.6 −1.0 −1.0 −1.1
2	ZINC000020530348	−14.187	ALA 74 ALA 74 ALA 74 ASN 94	−6.1 −2.7 −0.6 −1.1
3	ZINC000035239931	−13.211	ALA 74 ARG 117 LYS 69	−3.0 −1.3 −0.6
4	ZINC000021883347	−10.811	ASN 94 LYS 69	−1.3 −6.3
5	ZINC000012630694	−9.337	GLN 72 HIS 73	−5.3 −0.7
6	ZINC000012631011	−8.112	LYS 69	−7.8

## Data Availability

Not applicable.

## References

[1] Freney J., Brun Y., Bes M., Meugnier H., Grimont F., Grimont P.A.D., Nervi C., Fleurette J. (1988). Staphylococcus lugdunensis sp. nov. and Staphylococcus schleiferi sp. nov., Two Species from Human Clinical Specimens. Int. J. Syst. Evol. Microbiol..

[2] Jones R.M., Jackson M.A., Ong C., Lofland G.K. (2002). Endocarditis is caused by Staphylococcus lugdunensis. Pediatr. Infect. Dis. J..

[3] Kaabia N., Scauarda D., Lena G., Drancourt M. (2002). Molecular identification of Staphylococcus lugdunensis in a patient with meningitis. J. Clin. Microbiol..

[4] Ainoda Y., Takeshita N., Hase R., Mikawa T., Hosokawa N., Kawamura I., Ohmagari N., Kurai H., Abe M., Kimura M. (2016). Multicenter study of the clinical presentation of *Staphylococcus lugdunensis* bacteremia in Japan. Jpn. J. Infect. Dis..

[5] Zinkernagel A.S., Zinkernagel M.S., Elzi M.V., Genoni M., Gubler J., Zbinden R., Mueller N.J. (2008). Significance of Staphylococcus lugdunensis bacteremia: Report of 28 cases and review of the literature. Infection.

[6] Argemi X., Martin V., Loux V., Dahyot S., Lebeurre J., Guffroy A., Martin M., Velay A., Keller D., Prévost G. (2017). Whole-Genome Sequencing of Seven Strains of Staphylococcus lugdunensis Allows Identification of Mobile Genetic Elements. Genome Biol. Evol..

[7] Fujita S.-I., Senda Y., Iwagami T., Hashimoto T. (2005). Rapid Identification of Staphylococcal Strains from Positive-Testing Blood Culture Bottles by Internal Transcribed Spacer PCR Followed by Microchip Gel Electrophoresis. J. Clin. Microbiol..

[8] Frank K.L., Del Pozo J.L., Patel R. (2008). From clinical microbiology to infection pathogenesis: How daring to be different works for Staphylococcus lugdunensis. Clin. Microbiol. Rev..

[9] Tse H., Tsoi H.W., Leung S.P., Lau SK P., Woo PC Y., Yuen K.Y. (2010). Complete genome sequence of Staphylococcus lugdunensis strain HKU09-01. J. Bacteriol..

[10] Frank K.L., Patel R. (2007). Poly-N-acetylglucosamine is not a major component of the extracellular matrix in biofilms formed by icaADBC-positive Staphylococcus lugdunensis isolates. Infect. Immun..

[11] Tee W.S., Soh S.Y., Lin R., Loo L.H. (2003). Staphylococcus lugdunensis carrying the mecA gene causes catheter-associated bloodstream infection in a premature neonate. J. Clin. Microbiol..

[12] Al-Charrakh A.H., Obayes M.H. (2014). First record of isolation and characterization of methicillin-resistant Staphylococcus lugdunensis from clinical samples in Iraq. Biomed. Res. Int..

[13] Yen T.Y., Sung Y.J., Lin H.C., Peng C.T., Tien N., Hwang K.P., Lu J.J. (2016). The emergence of oxacillin-resistant Staphylococcus lugdunensis carrying staphylococcal cassette chromosome mec type V in central Taiwan. J. Microbiol. Immunol. Infect..

[14] Barh D., Tiwari S., Jain N., Ali A., Santos A., Misra A.N., Azevedo V., Kumar A. (2011). In Silico Subtractive Genomics for Target Identification in Human Bacterial Pathogens. Drug Dev. Res..

[15] Sakharkar M.K., Chow V.T., Kangueane P. (2004). Distributions of exons and introns in the human genome. In Silico Biol..

[16] Simeone R., Bottai D., Brosch R. (2009). ESX/type VII secretion systems and their role in host-pathogen interaction. Curr. Opin. Microbiol..

[17] Rahman A., Noore S., Hasan A., Ullah R., Rahman H., Hossain A., Ali Y., Islam S. (2014). Identifying potential drug targets by subtractive genome analysis of Bacillus anthracis A0248: An in silico approach. Comput. Biol. Chem..

[18] Chong C.E., Lim B.S., Nathan S., Mohamed R. (2006). In silico analysis of Burkholderia pseudomallei genome sequence for potential drug targets. In Silico Biol..

[19] Uddin R., Saeed K. (2014). Identifying and characterizing potential drug targets by subtractive genome analyses of methicillin-resistant Staphylococcus aureus. Comput. Biol. Chem..

[20] Rahman N., Shah M., Muhammad I., Khan H., Imran M. (2021). Genome-wide core proteome analysis of Brucella melitensis strains for potential drug target prediction. Mini-Rev. Med. Chem..

[21] Kumar V., Yadav C.S., Singh S., Goel S., Ahmed R.S., Gupta S., Grover R.K., Banerjee B.D. (2010). CYP 1A1 polymorphism and organochlorine pesticides levels in the aetiology of prostate cancer. Chemosphere.

[22] Huang Y., Niu B., Gao Y., Fu L., Li W. (2010). CD-HIT Suite: A web server for clustering and comparing biological sequences. Bioinformatics.

[23] Rahman N., Muhammad I., Nayab G.E., Khan H., Filosa R., Xiao J., Hassan S.T. (2019). In-silico subtractive proteomic analysis approach for therapeutic targets in MDR Salmonella enterica subsp. enterica serovar Typhi str. CT18. Curr. Top. Med. Chem..

[24] Taha M., Ismail N.H., Imran S., Wadood A., Rahim F., Saad S.M., Khan K.M., Nasir A. (2016). Synthesis, molecular docking and α-glucosidase inhibition of 5-aryl-2-(6′-nitrobenzofuran-2′-yl)-1,3,4-oxadiazoles. Bioorg. Chem..

[25] Moriya Y., Itoh M., Okuda S., Yoshizawa A.C., Kanehisa M. (2007). KAAS: Automatic genome annotation and pathway reconstruction server. Nucleic Acids Res..

[26] Mondal S.I., Ferdous S., Jewel N.A., Akter A., Mahmud Z., Islam M.M., Afrin T., Karim N. (2015). Identifying potential drug targets by subtractive genome analysis of Escherichia coli O157:H7: An in silico approach. Adv. Appl. Bioinform. Chem..

[27] Yoon S.H., Park Y.-K., Kim J.F. (2015). PAIDB v2.0: Exploration and analysis of pathogenicity and resistance islands. Nucleic Acids Res..

[28] Knox C., Law V., Jewison T., Liu P., Ly S., Frolkis A., Pon A., Banco K., Mak C., Wishart D.S. (2011). DrugBank 3.0: A comprehensive resource for ‘omics’ research on drugs. Nucleic Acids Res..

[29] Guarner F., Malagelada J.R. (2003). Gut flora in health and disease. Lancet.

[30] Sears C.L. (2005). A dynamic partnership: Celebrating our gut flora. Anaerobe.

[31] Keely S.P., Fischer J.M., Cushion M.T., Stringer J.R. (2004). Phylogenetic identification of Pneumocystis murina sp. nov., a new species in laboratory mice. Microbiology.

[32] Wu G.D., Lewis J.D. (2013). Analysis of the human gut microbiome and association with disease. Clin. Gastroenterol. Hepatol..

[33] Gaur V., Wyatt H.D.M., Komorowska W., Szczepanowski R.H., de Sanctis D., Gorecka K.M., West S.C., Nowotny M. (2015). Structural and Mechanistic Analysis of the Slx1-Slx4 Endonuclease. Cell Rep..

[34] Wiederstein M., Sippl M.J. (2007). ProSA-web: Interactive web service for recognizing errors in three-dimensional structures of proteins. Nucleic Acids Res..

[35] Wang J., Wolf R.M., Caldwell J.W., Kollman P.A., Case D.A. (2004). Development and testing of a general amber force field. J. Comput. Chem..

[36] Yin L.L., Xu J.K., Wang X.J., Gao S.Q., Lin Y.W. (2020). Molecular Dynamics Simulation and Kinetic Study of Fluoride Binding to V21C/V66C Myoglobin with a Cytoglobin-like Disulfide Bond. Int. J. Mol. Sci..

[37] Chen S., He Y., Geng Y., Wang Z., Han L., Han W. (2021). Molecular Dynamic Simulations of Bromodomain and Extra-Terminal Protein 4 Bonded to Potent Inhibitors. Molecules.

[38] Rahman N., Ajmal A., Ali F., Rastrelli L. (2020). Core proteome mediated therapeutic target mining and multi-epitope vaccine design for Helicobacter pylori. Genomics.

[39] Kanehisa M., Furumichi M., Tanabe M., Sato Y., Morishima K. (2017). KEGG: New perspectives on genomes, pathways, diseases and drugs. Nucleic Acids Res..

[40] Willing B.P., Russell S.L., Finlay B.B. (2011). Shifting the balance: Antibiotic effects on host-microbiota mutualism. Nat. Rev. Microbiol..

[41] Kufareva I., Abagyan R. (2012). Methods of protein structure comparison. Methods Mol. Biol..

[42] Sargsyan K., Grauffel C., Lim C. (2017). How Molecular Size Impacts RMSD Applications in Molecular Dynamics Simulations. J. Chem. Theory Comput..

[43] Sabiu S., Balogun F.O., Amoo S.O. (2021). Phenolics Profiling of *Carpobrotus edulis* (L.) N.E.Br. and Insights into Molecular Dynamics of Their Significance in Type 2 Diabetes Therapy and Its Retinopathy Complication. Molecules.

[44] Lobanov M.Y., Bogatyreva N.S., Galzitskaya O.V. (2008). The radius of gyration is an indicator of protein structure compactness. Mol. Biol..

[45] Emmanuel I.A., Olotu F., Agoni C., Soliman M.E. (2019). Broadening the horizon: Integrative pharmacophore-based and cheminformatics screening of novel chemical modulators of mitochondria ATP synthase towards interventive Alzheimer’s disease therapy. Med. Hypotheses.

[46] Basharat Z., Jahanzaib M., Rahman N. (2021). Therapeutic target identification via differential genome analysis of antibiotic-resistant Shigella sonnei and inhibitor evaluation against a selected drug target. Infect. Genet. Evol..

